# Development of a local antibiogram for a teaching hospital in Ghana

**DOI:** 10.1093/jacamr/dlad024

**Published:** 2023-03-27

**Authors:** Cornelius C Dodoo, Hayford Odoi, Adelaide Mensah, Karikari Asafo-Adjei, Ruth Ampomah, Lydia Obeng, Jonathan Jato, Araba Hutton-Nyameaye, Thelma A Aku, Samuel O Somuah, Emmanuel Sarkodie, Emmanuel Orman, Kwadwo A Mfoafo, Inemesit O Ben, Eneyi E Kpokiri, Fatima Abba, Yogini H Jani

**Affiliations:** School of Pharmacy, University of Health and Allied Sciences, Ho, Ghana; School of Pharmacy, University of Health and Allied Sciences, Ho, Ghana; School of Pharmacy, University of Health and Allied Sciences, Ho, Ghana; Laboratory Department, Ho Teaching Hospital, Ho, Ghana; School of Pharmacy, University of Health and Allied Sciences, Ho, Ghana; School of Pharmacy, University of Health and Allied Sciences, Ho, Ghana; School of Pharmacy, University of Health and Allied Sciences, Ho, Ghana; School of Pharmacy, University of Health and Allied Sciences, Ho, Ghana; School of Pharmacy, University of Health and Allied Sciences, Ho, Ghana; School of Pharmacy, University of Health and Allied Sciences, Ho, Ghana; Pharmacy Department, Kwame Nkrumah University of Science and Technology Teaching Hospital, Kumasi, Ghana; School of Pharmacy, University of Health and Allied Sciences, Ho, Ghana; School of Pharmacy, University of Health and Allied Sciences, Ho, Ghana; School of Pharmacy, University of Health and Allied Sciences, Ho, Ghana; Department of Clinical Research, Faculty of Infectious and Tropical Diseases, London School of Hygiene and Tropical Medicine, London, UK; Health Protection Operations Division, UK Health Security Agency, London, UK; Centre for Medicines Optimisation Research and Education, University College London Hospitals NHS Trust & UCL School of Pharmacy, London, UK; Centre for Medicines Optimisation Research and Education, University College London Hospitals NHS Trust & UCL School of Pharmacy, London, UK

## Abstract

**Background:**

Antimicrobial resistance threatens adequate healthcare provision against infectious diseases. Antibiograms, combined with patient clinical history, enable clinicians and pharmacists to select the best empirical treatments prior to culture results.

**Objectives:**

To develop a local antibiogram for the Ho Teaching Hospital.

**Methods:**

This was a retrospective cross-sectional study, using data collected on bacterial isolates from January–December 2021. Samples from urine, stool, sputum, blood, and cerebrospinal fluid (CSF) were considered as well as, aspirates and swabs from wound, ears and vagina of patients. Bacteria were cultured on both enrichment and selective media including blood agar supplemented with 5% sheep blood and MacConkey agar, and identified by both the VITEK 2 system and routine biochemical tests. Data on routine culture and sensitivity tests performed on bacterial isolates from patient samples were retrieved from the hospital’s health information system. Data were then entered into and analysed using WHONET.

**Results:**

In all, 891 pathogenic microorganisms were isolated from 835 patients who had positive culture tests. Gram-negative isolates accounted for about 77% of the total bacterial species. *Escherichia coli* (246), *Pseudomonas* spp. (180), *Klebsiella* spp. (168), *Citrobacter* spp. (101) and *Staphylococcus* spp. (78) were the five most isolated pathogens. Most of the bacterial isolates showed high resistance (>70%) to ampicillin, piperacillin, ceftazidime, ceftriaxone, cefotaxime, penicillin G, amoxicillin, amoxicillin/clavulanic acid, ticarcillin/clavulanic acid and trimethoprim/sulfamethoxazole.

**Conclusions:**

The isolates from the various samples were not susceptible to most of the antibiotics used in the study. The study reveals the resistance patterns of *E. coli* and *Klebsiella* spp. to some antibiotics on the WHO ‘Watch’ and ‘Reserve’ lists. Using antibiograms as part of antimicrobial stewardship programmes would optimize antibiotic use and preserve their efficacy.

## Introduction

Antimicrobial resistance (AMR) is a global problem with many causes including inappropriate prescribing of antimicrobials.^1,2^ A key strategy to address AMR is by employing a targeted approach to treatment, to reduce indiscriminate prescribing, thereby conserving the efficacy of antimicrobials. This presents a challenge, especially in the developing world due to limited availability of efficient diagnostic measures, making it difficult to know the true burden of AMR.^3^ In Ghana, antimicrobial therapy constitutes a major form of treatment in all healthcare facilities.^4,5^ However, treatment is mainly empirical due to a relative lack of appropriate laboratory and diagnostic facilities for culture and sensitivity testing of bacteria in most healthcare facilities. Even where laboratory facilities are available, culture and sensitivity tests performed present additional medical costs to the patient and may often not be recommended.^6^

An antibiogram, a periodic summary of antimicrobial susceptibilities of bacterial isolates submitted by a hospital’s clinical microbiology laboratory, can serve as the primary source of validated data to be used by clinicians to assess local antimicrobial susceptibility patterns of pathogens and guide empirical therapy or selection of antimicrobials.^7–9^ Local, regional and national antibiogram data generated from healthcare facilities are key in the monitoring of trends in AMR and guiding the selection of effective antibiotics for empirical therapy.^10,11^ The development of local hospital antibiograms can therefore serve as the foundation for AMR surveillance, clinical decision support for rational antimicrobial use, and identify areas for intervention by antimicrobial stewardship (AMS) programmes.^12^

Data on the AMR profiles of clinically relevant pathogenic bacterial isolates like *Neisseria gonorrhoeae* and *Shigella* spp. have been reported from some hospitals in Ghana.^13,14^ However, there are limited comprehensive institutional data on susceptibility patterns of common pathogens for most hospitals in Ghana and sub-Saharan Africa.^3,15^ Furthermore, there are no documented data on the bacterial isolates and antibiotic resistance profiles at the Ho Teaching Hospital (HTH). This could lead to the irrational selection of antimicrobials for empirical therapy, which will further compound the problem of AMR within the facility. The objective of this study was therefore to develop a local antibiogram for HTH through retrospective analysis of laboratory data.

## Methods

### Study design and study site

This was a 12–month retrospective cross-sectional study conducted in HTH, a tertiary healthcare facility located in Ho, Ghana. The hospital has a bed capacity of about 300 and 14 wards. It is the main referral facility in the Volta Region and has five clinical departments, namely, internal medicine, surgical, obstetrics & gynaecology, child health and public health. The facility also has a microbiology laboratory and an AMS committee.

### Laboratory techniques

As part of routine care and practice, culture and susceptibility tests were performed on bacterial isolates from urine, stool, sputum and blood samples obtained from both outpatients and inpatients who visited the hospital within the study period. Swabs from wound, ear and vagina of patients were also subjected to these tests. Specimens that had been collected, processed and analysed in the microbiology unit of the HTH employing HTH guidelines for culture and microbial identification were considered. Bacteria were cultured on both enrichment and selective media including blood agar supplemented with 5% sheep blood for Gram-positive cocci and MacConkey agar for Gram-negative bacilli. Isolates were identified by both the VITEK 2 system and routine biochemical tests including catalase and coagulase tests for Gram-positive cocci. Antimicrobial susceptibility was performed using the Kirby–Bauer disc diffusion technique following CLSI 2021 (31st edition) standards.

All the culture-positive samples were included in the study, and repeat isolates from the same person were excluded in order to avoid duplication. The bacterial isolates identified in these samples and their sensitivity or resistance to antimicrobials were recorded in the hospital’s information management system, Lightwave Health Information Management System (LHIMS), as susceptible, intermediate or resistant.

### Data collection and analysis

Routinely collected data on all isolates reported on the LHIMS from January 2021 to December 2021 were extracted/entered and organized into a Microsoft Excel 2022 datasheet. This was then exported and analysed using WHONET (version 5.6), a Windows-based database software package for the management of microbiology laboratory data and the analysis of antimicrobial susceptibility test results.

### Antibiogram development

The aggregated data from WHONET produced susceptibility percentages for every organism. Organisms with fewer than 30 isolates were initially excluded, given the potential for diminished accuracy. The list was then reviewed to include other clinically important pathogenic microorganisms, ESKAPE pathogens (*Enterococcus faecium*, *Staphylococcus aureus*, *Klebsiella pneumoniae*, *Acinetobacter baumannii*, *Pseudomonas aeruginosa*, *Enterobacter* spp.) that did not have 30 isolates.^16^ The antibiotics included in the antibiogram were narrowed to antibiotics in the WHO Watch and Reserve categories that were tested in the hospital.^17^

### Ethics

Ethical clearance (UHAS-REC A.5[3]21-22) was obtained from the Research Ethics Committee of the University of Health and Allied Sciences, Ho. Permission was also sought from the hospital to carry out the research.

## Results

A total of 891 pathogenic microorganisms were isolated from 835 patient samples with positive culture tests (Table 1). Gram-negative isolates accounted for about 77% of the total bacterial species. *Escherichia coli* (246), *Pseudomonas* spp. (180), *Klebsiella* spp. (168), *Citrobacter* spp. (101) and *Staphylococcus* spp. (78) were the top five commonly isolated pathogens. Of the total *Pseudomonas* spp. isolated, 114 were identified to be *P. aeruginosa* while 54 *Klebsiella oxytoca* and 13 *K. pneumoniae* isolates were identified out of the total *Klebsiella* spp. isolated. 34 *Citrobacter koseri* isolates and 56 *S. aureus* isolates were identified from the total *Citrobacter* spp. and *Staphylococcus* spp., respectively.

**Table 1.** Prevalence of isolates per microorganism

Microorganism	Number of isolates (*n*)	Percentage (%)
*E. coli*	246	27.61
*Pseudomonas* spp.	180	20.20
*Klebsiella* spp.	168	18.86
*Citrobacter* spp.	101	11.34
*Staphylococcus* spp.	78	8.75
*Enterobacter* spp.	21	2.36
*Acinetobacter* spp.	20	2.24
*Enterococcus* spp.	15	1.68
*Proteus vulgaris*	13	1.46
*P. mirabilis*	12	1.35
*Providencia* spp.	10	1.12
*Serratia marcescens*	7	0.79
*Morganella morganii* ssp. *morganii*	5	0.56
*Moraxella catarrhalis*	4	0.45
*Salmonella* spp.	4	0.45
*Shigella* spp.	3	0.34
*Francisella tularensis* ssp. *tularensis*	1	0.11
*Micrococcus luteus*	1	0.11
*Neisseria meningitidis*	1	0.11
*Streptococcus pyogenes*	1	0.11

Urinary tract infections (UTIs; 477), wound (184) and ear (115) infections (Table S1) were the most commonly reported during the study period. *E. coli* (204) and *Klebsiella* spp. (62) were responsible for most of the UTIs while *Pseudomonas* spp. (77) accounted for most of the ear infections. For the wound infections, however, there was a near equal number of isolates for *E. coli* (26) and *S. aureus* (23) while 46 *Pseudomonas* spp. were isolated. A complex diversity of microbial pathogens was associated with urine (21), wound (21), ear (16), blood (15) and vaginal (13) samples obtained from patients (Table S1).

**Figure 1.**
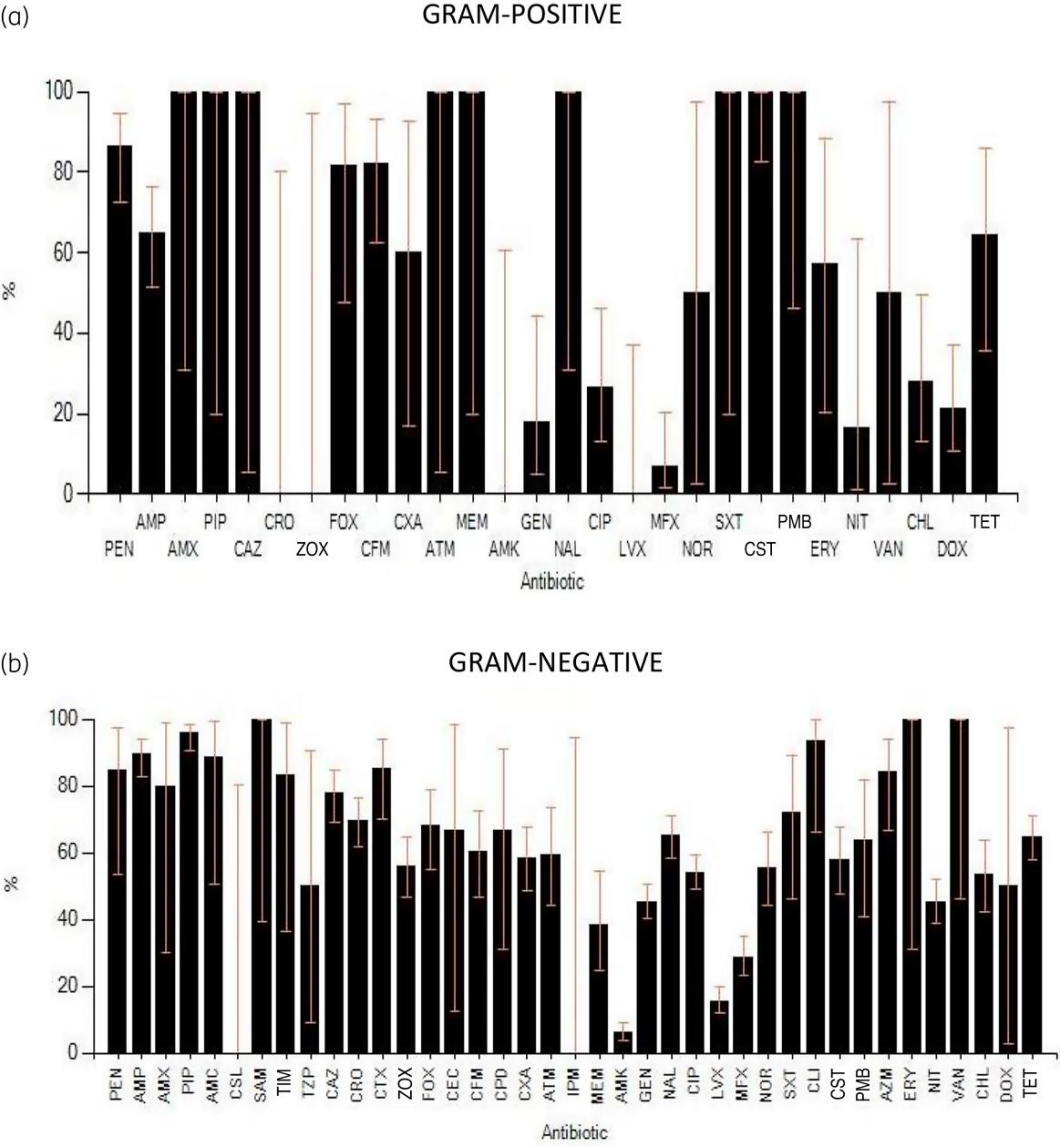
Resistance pattern of (a) Gram-positive and (b) Gram-negative bacteria. AMC, amoxicillin/clavulanic acid, 20 μg; AMK, amikacin 30 μg; AMP, ampicillin 10 μg; AMX, amoxicillin 30 μg; ATM, aztreonam 30 μg; AZM, azithromycin 15 μg; CAZ, ceftazidime 30 μg; CEC, cefaclor 30 μg; CFM, cefixime 5 μg; CHL, chloramphenicol 30 μg; CIP, ciprofloxacin 5 μg; CLI, clindamycin 2 μg; CST, colistin 10 μg; CPD, cefpodoxime 10 μg; CPO, cefpirome 30 μg; CRO, ceftriaxone 30 μg; CSL, cefoperazone/sulbactam 30 μg; CTX, cefotaxime 30 μg; CXM, cefuroxime 30 μg; ZOX, ceftizoxime 30 μg; DOX, doxycycline 30 μg; ERY, erythromycin 15 μg; FOX, cefoxitin 30 μg; GEN, gentamicin 10 μg; IPM, imipenem 10 μg; LVX, levofloxacin 5 μg; MEM, meropenem 10 μg; MFX, moxifloxacin 5 μg; NAL, nalidixic acid 30 μg; NIT, nitrofurantoin 300 μg; NOR, norfloxacin 10 μg; PEN, penicillin G 10 μg; PIP, piperacillin 100 μg; PMB, polymyxin B 300 μg; SAM, ampicillin/sulbactam 10 μg; SXT, trimethoprim/sulfamethoxazole 1.2 μg; TCC, ticarcillin/clavulanic acid 75 μg; TET, tetracycline 30 μg; TMP, trimethoprim 5μg; TZP, piperacillin/tazobactam 100 μg; VAN, vancomycin 30 μg.

The resistance of bacterial isolates to the antibiotics tested were expressed as percentages (Figure 1). A total of 44 different antibiotics were tested. However, not all the antibiotics were tested on all the microbes by the Infectious Diseases Laboratory at HTH. This decision was informed mainly by the availability of antibiotic discs and the inventory of antibiotics at the hospital. The bacterial isolates showed high resistance (>70%) to ampicillin, piperacillin, ceftazidime, ceftriaxone, cefotaxime, penicillin G, amoxicillin, co-amoxiclav, ampicillin/sulbactam, ticarcillin/clavulanic acid, cefpirome and trimethoprim/sulfamethoxazole. With the exception of ampicillin, piperacillin, ceftazidime, and ceftriaxone, which were tested against more than 100 isolates, all other antibiotics were tested against less than 20 isolates and cefotaxime was tested against 32 isolates. Inference was therefore made considering the number of isolates tested against an antibiotic of interest. Amikacin, levofloxacin and moxifloxacin showed the highest activity against all bacterial isolates.

An antibiogram was generated using the percentage susceptibility of the pathogens indicated in Table 1. The susceptibility of these organisms to selected antibiotics on the WHO Watch and Reserve list are shown in Table S2. Microorganisms with susceptibility greater than 70% for a specific test antibiotic were regarded as highly susceptible while those showing susceptibilities between 40% and 69%, and less than 40% were regarded as intermediate and resistant, respectively.

*E. coli*, the most abundant Gram-negative isolate, showed very poor susceptibility to piperacillin, piperacillin/tazobactam, ceftriaxone, ceftazidime, ceftizoxime, cefotaxime, aztreonam and ciprofloxacin. However, levofloxacin showed a markedly pronounced activity (78%) against most *E. coli* isolates. *Pseudomonas* spp. and *P. aeruginosa* were susceptible to azithromycin (100% and 83%, respectively) and moxifloxacin (73% and 86%, respectively). *S. aureus* and *Enterococcus* spp. were more susceptible to levofloxacin (100% for both microbes) and moxifloxacin (82% and 80%, respectively). Generally, piperacillin, ceftriaxone, cefuroxime, ceftazidime, ceftizoxime, cefixime, cefoxitin, cefpodoxime, cefpirome, cefotaxime, cefaclor and aztreonam showed very poor activity against all the microbial isolates tested.

## Discussion

The use of antibiograms to guide the selection of empirical antibiotic therapy for a suspected microbial infection is a well-established practice.^9,15,18^ To the best of our knowledge, this is the first comprehensive study that describes antibiogram data in HTH. Out of the total pathogenic isolates, *E. coli* (246), *Pseudomonas* spp. (180) and *Klebsiella* spp. (168) were the most commonly isolated microbes from routine tests conducted in HTH from the different patient samples during the study period. Other pathogens like *Citrobacter* spp. and *S. aureus* were isolated more than 30 times. Most of these pathogenic microbes were obtained as a result of UTIs (468) and wound (182) and ear (115) infections. UTIs and wounds have been reported to be among the commonest sources typically presenting with pathogens.^19–21^

The highly diversified nature of microbial pathogens associated with urine, vagina, wound, ear and blood samples suggests a polymicrobial complexity to associated bacteraemia, UTIs, and wound and ear infections. The high number of cultures showing *E. coli* and *Klebsiella* spp. did not come as surprise due to the equally high number of UTIs reported during the period. Out of the 477 UTIs, *E. coli* and *Klebsiella* spp. were isolated in 204 and 106 instances, respectively. These findings were similar to other reports from referral hospitals, where a high prevalence of these two pathogens were obtained for UTIs.^22–24^*E. coli* is known to be the commonest cause of UTI, with other Enterobacteriaceae like *Klebsiella* spp. also implicated in most of these infections.^20,25^

Wound infections, the second highest source, had *E. coli* (26), *Pseudomonas* spp. (46) and *S. aureus* (23) isolated often (Table S1). Globally, bacterial infections of wounds are among the leading causes of morbidity and mortality and are regarded as one of the commonest nosocomial infections.^26^*S. aureus*, *P. aeruginosa* and other coliforms (23%) have been reported to be predominant in acute wounds, while chronic wounds usually had *Proteus mirabilis*, *Enterococcus* spp. and *E. coli.*^21,23,27^ In this work, the nature of the wound was not taken into consideration during data extraction.

The prevalence of ear infections has been reported to be on the rise in developing countries, with bacteria being major causes of these. Although primarily a disease of infants and young children, adults can also be affected by ear infections.^28^ Complications like meningitis and brain abscess could arise if infection is not properly managed as a result of the causative organisms being resistant to treatment.^29^ In this study *Pseudomonas* spp. were responsible for almost 70% of ear infections; *S. aureus* was identified in about 10% of the cases. Similar findings have been reported where *S. aureus* and *P. aeruginosa* were predominant causes of ear infections.^29,30^ Generally, ear infections are mainly caused by microbes found on the skin of the external ear that gain access to the middle ear through perforation.^28^

Antibiotic susceptibility testing of the pathogens to the different antibiotics revealed percentage resistance above 70% for most of the antibiotics tested. Taking into consideration the total number of samples tested per antibiotic, ampicillin, piperacillin, ceftriaxone and ceftazidime showed the least activity against the pathogens. It is interesting to note that all these are β-lactam antibiotics, indicating the need to intensify stewardship activities in this regard. Globally, the increase in acquired resistance to β-lactams and ESBL-producing bacteria is one of great concern.^21^

Further emphasis was placed on obtaining the resistance profile of the commonly isolated organisms (more than 30 isolates) as well as commonly isolated clinically relevant microbes (i.e. other microbes of the ESKAPE group with less than 30 isolates). Other ESKAPE pathogens were considered since these microbes are considered the six most common MDR pathogens globally.^16^ All these pathogens were Gram-negative organisms except *S. aureus* and *Enterococcus* spp.

The epidemiologically significant Gram-negative pathogens indicated high resistance to most of the β-lactams tested (piperacillin, ceftriaxone, cefuroxime, ceftazidime and ceftizoxime) . Only *Acinetobacter* spp. were susceptible to ceftriaxone, but there was just one isolate; hence it was difficult to draw any meaningful inference. The high resistance of the Gram-negative isolates, particularly in the Enterobacteriaceae genera, to the third-generation cephalosporins suggests notable alert levels of possible circulating ESBL-producing Enterobacteriaceae in the tertiary care facility (Figure 1). Resistance of Gram-negative rods to these β-lactam antibiotics is a recent phenomenon that has been reported.^15,20,31–33^ Studies from sub-Saharan Africa reveal high rates of ESBL production or resistance, especially to third-generation cephalosporins.^22,23,31,34,35^ Similarly, a study done at Korle-Bu Teaching Hospital, Ghana found high levels of ESBL-producing enterobacteria as a significant cause of infections and resistance at the hospital.^36^

Although available literature supports low susceptibility of *E. coli* and *K. pneumoniae* to cephalosporins,^22,37^ these alarming findings are worthy of intensive stewardship activities. A noteworthy observation was the high resistance by the Gram-negative rods to ceftazidime, a drug on the WHO Watch list. *E. coli* for instance had a resistance of 75% to ceftriaxone, an antibiotic also on the WHO Watch list. Third-generation cephalosporins are commonly used antibiotics in UTIs; however, with the growing high resistance to these antibiotics, prudent use of these may be needed.^38,39^ Resistance of *Pseudomonas* spp. to ceftazidime and other third-generation cephalosporins has also been reported at other tertiary healthcare facilities in Rwanda and Tanzania.^23,32^

Colistin, the most tested polymyxin, had resistance developed to it, especially by *S. aureus* and *P. aeruginosa.* (Table S3, available as Supplementary data at *JAC-AMR* Online). As one of the antibiotics on the WHO Reserve list, these microbes exhibiting high resistance to this antibiotic are a great concern.^17^ A recent study in Egypt found colistin to be one of their most effective antibiotics, with a susceptibility of at least 90% for Gram-negative rods like *E. coli*, *Klebsiella* spp. and *A. baumannii*. Interestingly, they reported 79.4% susceptibility for *P. aeruginosa* in their work.^37^ This indicates that this drug is still an effective treatment option in Africa and efforts to ensure its continued efficacy in HTH facility is warranted.

The quinolones frequently tested during the period were ciprofloxacin, levofloxacin and moxifloxacin. Levofloxacin showed good activity against the pathogens indicated. Moxifloxacin was also effective in most of the microbes with the exception of *C. koseri* (25%). Ciprofloxacin also had good susceptibility except for *C. koseri*, *E. coli* and *K. oxytoca*, where less than 40% susceptibility was obtained. Increasing resistance to fluoroquinolones has also been reported, especially in *E. coli* and *K. pneumoniae.*^15,32^ From our work, the quinolones remain the class of antibiotic with high efficacy against pathogens in the facility, and effort needs to be put in place to protect these.

A limitation in our study was the fact that the number of isolates of some pathogens or antibiotics tested on some isolates was small. Such organisms were not commented on, even though these could also be pathogens or antibiotics that awareness and stewardship activities should target.

### Conclusions

The general antibiotic susceptibility pattern of the study isolates shows an overall high drug resistance to many routinely tested antibiotics. Resistance patterns obtained from the data revealed a trend of some antibiotics on the WHO Watch and Reserve lists gradually losing efficacy towards some of the commonly isolated pathogens, especially *E. coli* and *Klebsiella* spp. These findings thus emphasize the need for a robust AMS programme that can implement interventions to improve antibiotic use and preserve the efficacy of antibiotics.

Based on the findings of this study, ceftazidime, ceftriaxone and colistin should be key targets of AMS in HTH. Considering these antibiotics are on the WHO Watch and Reserve lists of antibiotics, they should be used with caution or as last-resort options.

## Supplementary Material

dlad024_Supplementary_Data

## Supplementary data

Tables S1–S3 are available as Supplementary data at *JAC-AMR* Online.
