# Supplementary material for: Development of a local antibiogram for a teaching hospital in Ghana
Source: JAC Antimicrob Resist. 2023 Mar 27;5(2):dlad024. doi: 10.1093/jacamr/dlad024 (PMC10041059; doi:10.1093/jacamr/dlad024)
Supplement: dlad024_Supplementary_Data [file dlad024_supplementary_data.docx]

**Development of a local antibiogram for a Teaching Hospital in Ghana**

Cornelius C. DODOO^1*^, Hayford ODOI^1^, Adelaide MENSAH^1^, Karikari ASAFO-ADJEI^2^, Ruth AMPOMAH^1^, Lydia OBENG^1^, Jonathan JATO^1^, Araba HUTTON-NYAMEAYE^1^, Thelma A. AKU^1^, Samuel O. SOMUAH^1^, Emmanuel SARKODIE^3^, Emmanuel ORMAN^1^, Kwadwo A. MFOAFO^1^, Inemesit O. BEN^1^, Eneyi E. KPOKIRI^4^, Fatima ABBA^5,6^, Yogini H. JANI^6^

^1^ School of Pharmacy, University of Health and Allied Sciences, Ho, Ghana

^2^ Ho Teaching Hospital, Ho, Ghana

^3^ Kwame Nkrumah University of Science and Technology Teaching Hospital, Kumasi, Ghana

^4^ Department of Clinical Research, Faculty of Infectious and Tropical Diseases, London School of Hygiene and Tropical Medicine, London, UK

^5^ UK Health Security Agency, London, United Kingdom.

^6^ Centre for Medicines Optimisation Research and Education, University College London Hospitals NHS Trust & UCL School of Pharmacy, London, UK

*Corresponding author’s email: cdodoo@uhas.edu.gh

Table S1: Distribution of bacterial isolates in samples

| **Sample Code** | **Number of isolates** | *Acinetobacter* spp | *Moraxella catarrhalis* | *Citrobacter koseri* | *Citrobacter freundii* | *Citrobacter* spp | *Escherichia coli* | *Enterobacter sp* | *Enterococcus* spp | *Francisella tularensis* | *Klebsiella* spp | *Klebsiella oxytoca* | *Klebsiella pneumoniae* | *Klebsiella pneumoniae ss. Rhinoscleromatis* | *Morganella morganii* | *Neisseria meningitidis* | *Pseudomonas aeruginosa* | *Proteus mirabilis* | *Providencia rettgeri* | *Providencia* spp | *Pseudomonas* spp | *Proteus vulgaris* | *Salmonella* spp | *Staphylococcus saprophyticus* | *Salmonella typhi* | *Staphylococcus aureus* | *Staphylococcus, coagulase-negative* | *Staphylococcus epidermidis* | *Shigella sp*. | *Serratia marcescens* | *Streptococcus pyogenes* |
| --- | --- | --- | --- | --- | --- | --- | --- | --- | --- | --- | --- | --- | --- | --- | --- | --- | --- | --- | --- | --- | --- | --- | --- | --- | --- | --- | --- | --- | --- | --- | --- |
| **Urine** | 477 | 7 |  | 22 | 4 | 34 | 204 | 12 | 10 |  | 62 | 41 | 2 | 1 |  |  | 29 | 1 |  | 7 | 16 | 4 |  | 6 |  | 11 |  |  | 1 | 2 |  |
| **Wound** | 184 | 6 |  | 10 | 2 | 12 | 26 | 3 | 2 |  | 14 | 10 | 4 |  | 3 |  | 26 | 10 | 1 |  | 20 | 6 | 1 |  |  | 23 |  | 1 | 1 | 3 |  |
| **Ear** | 115 | 1 |  |  |  | 4 | 2 | 3 |  |  | 7 |  | 1 |  | 1 |  | 54 |  | 1 | 1 | 23 | 3 |  | 1 |  | 11 |  | 1 | 1 |  |  |
| **Vagina** | 39 | 1 |  | 2 |  | 4 | 10 | 1 | 1 |  | 7 | 2 |  |  | 1 |  | 2 |  |  |  | 1 |  |  |  |  | 6 |  | 1 |  |  |  |
| **Blood** | 34 | 3 |  |  |  | 3 | 1 |  |  | 1 | 4 | 1 | 2 |  |  | 1 | 1 | 1 |  |  | 3 |  |  | 3 |  |  |  | 7 |  | 2 | 1 |
| **Sputum** | 17 | 2 | 2 |  |  | 4 |  |  |  |  | 4 |  | 2 |  |  |  | 2 |  |  |  | 1 |  |  |  |  |  |  |  |  |  |  |
| **Throat** | 7 |  | 2 |  |  |  |  | 2 | 2 |  | 1 |  |  |  |  |  |  |  |  |  |  |  |  |  |  |  |  |  |  |  |  |
| **Stool** | 5 |  |  |  |  |  | 1 |  |  |  | 1 |  |  |  |  |  |  |  |  |  |  |  | 1 |  | 2 |  |  |  |  |  |  |
| **Urethra** | 4 |  |  |  |  |  | 1 |  |  |  |  |  |  |  |  |  |  |  |  |  | 1 |  |  | 1 |  | 1 |  |  |  |  |  |
| **Aspirate** | 3 |  |  |  |  |  |  |  |  |  |  |  |  |  |  |  |  |  |  |  | 1 |  |  |  |  | 2 |  |  |  |  |  |
| **Umbilical cord** | 3 |  |  |  |  |  |  |  |  |  |  |  |  |  |  |  |  |  |  |  |  |  |  |  |  | 2 | 1 |  |  |  |  |
| **Pleural fluid** | 1 |  |  |  |  |  | 1 |  |  |  |  |  |  |  |  |  |  |  |  |  |  |  |  |  |  |  |  |  |  |  |  |
| **CSF** | 1 |  |  |  |  |  |  |  |  |  |  |  | 1 |  |  |  |  |  |  |  |  |  |  |  |  |  |  |  |  |  |  |
| **Semen** | 1 |  |  |  |  |  |  |  |  |  | 1 |  |  |  |  |  |  |  |  |  |  |  |  |  |  |  |  |  |  |  |  |

Table S2: An antibiogram based on WHO Aware classification

| Epidemiological significant pathogens | N |  | Piperacillin | Ceftriaxone | Cefuroxime | Ceftazidime | Ceftizoxime | Cefixime | Cefoxitin | Cefpodoxime | Cefpirome | Cefotaxime | Cefaclor | Aztreonam | Meropenem | Colistin | Polymyxin B | Azithromycin | Erythromycin | Ciprofloxacin | Levofloxacin | Norfloxacin | Moxifloxacin |
| --- | --- | --- | --- | --- | --- | --- | --- | --- | --- | --- | --- | --- | --- | --- | --- | --- | --- | --- | --- | --- | --- | --- | --- |
| *Acinetobacter* spp*.* | **20** | **n** |  |  |  |  | 6 |  | 6 |  |  |  |  |  |  |  |  |  |  | 7 | 5 |  | 10 |
|  |  | **%S** |  |  |  |  | 33***** |  | 33***** |  |  |  |  |  |  |  |  |  |  | **57** | *80* |  | **60** |
| *Citrobacter koseri* | **34** | **n** |  | 6 |  |  | 7 |  |  |  |  |  |  |  |  |  |  |  |  | 20 | 17 |  | 12 |
|  |  | **%S** |  | 33***** |  |  | 28***** |  |  |  |  |  |  |  |  |  |  |  |  | 30***** | *70* |  | 25***** |
| *Citrobacter* spp. | **61** | **n** | 14 | 17 | 6 | 9 | 12 | 5 |  |  |  |  |  |  |  | 5 |  |  |  |  | 29 | 6 | 22 |
|  |  | **%S** | 0***** | 23***** | 33***** | 0***** | 25***** | 20***** |  |  |  |  |  |  |  | **40** |  |  |  |  | *89* | **50** | 77 |
| *Enterobacter* sp*.* | **21** | **n** |  |  |  |  |  |  |  |  |  |  |  |  |  |  |  |  |  | 12 | 8 |  | 7 |
|  |  | **%S** |  |  |  |  |  |  |  |  |  |  |  |  |  |  |  |  |  | *66* | *100* |  | *85* |
| *Enterococcus* spp*.* | **15** | **n** |  |  |  |  |  |  |  |  |  |  |  |  |  |  |  |  |  | 10 | 6 |  | 5 |
|  |  | **%S** |  |  |  |  |  |  |  |  |  |  |  |  |  |  |  |  |  | **60** | *100* |  | *80* |
| *Escherichia coli* | **246** | **n** | 61 | 85 | 50 | 67 | 25 | 11 | 14 |  |  | 16 |  | 6 | 19 | 10 |  |  |  | 172 | 149 | 44 | 32 |
|  |  | **%S** | 2***** | 25***** | **44** | 19***** | 32***** | **45** | **42** |  |  | 13***** |  | 33***** | **52** | 70 |  |  |  | 37***** | *78* | **45** | **37** |
| *Klebsiella pneumoniae*  *ss. pneumoniae* | **12** | **n** |  |  |  |  |  |  |  |  |  |  |  |  |  |  |  |  |  |  |  |  | 7 |
|  |  | **%S** |  |  |  |  |  |  |  |  |  |  |  |  |  |  |  |  |  |  |  |  | *100* |
| *Klebsiella oxytoca* | **54** | **n** | 11 | 16 | 13 | 12 | 6 |  |  |  |  |  |  |  | 8 | 5 |  |  |  | 37 | 27 | 7 | 10 |
|  |  | **%S** | 19***** | 13***** | 23***** | 8***** | 0***** |  |  |  |  |  |  |  | **63** | **40** |  |  |  | 21***** | *70* | 14***** | **40** |
| *Klebsiella spp.* | **101** | **n** | 20 | 22 | 18 | 10 | 19 | 13 | 11 |  |  | 10 |  | 7 |  | 15 | 5 |  |  | 53 | 49 | 16 | 27 |
|  |  | **%S** | 0***** | 22***** | 27***** | 10***** | **42** | **46** | 27***** |  |  | 10***** |  | 14***** |  | **60** | 20***** |  |  | **58** | *79* | 44***** | *81* |
| *Pseudomonas aeruginosa* | **114** | **n** | 5 |  |  | 6 | 23 | 10 |  |  |  |  |  | 6 |  | 24 | 6 | 12 |  | 24 | 29 |  | 36 |
|  |  | **%S** | 0***** |  |  | 16***** | **60** | 0***** |  |  |  |  |  | 16***** |  | 12***** | 16***** | *83* |  | **41** | **48** |  | *86* |
| *Pseudomonas spp.* | **66** | **n** |  |  |  | 5 | 8 |  | 8 |  |  |  |  | 9 |  | 19 |  |  |  | 14 | 11 |  | 42 |
|  |  | **%S** |  |  |  | **40** | 38***** |  | **50** |  |  |  |  | 22***** |  | **42** |  |  |  | **50** | *72* |  | *73* |
| *Staphylococcus aureus ss. aureus* | **56** | **n** |  |  |  |  |  | 23 |  |  |  |  |  |  |  | 22 |  |  |  | 11 | 7 |  | 39 |
|  |  | **%S** |  |  |  |  |  | 8***** |  |  |  |  |  |  |  | 27***** |  |  |  | *90* | *100* |  | *82* |

WHO reserve list of antibiotics (Aztreonam, colistin, Polymyxin B) ; n: number of isolates tested per antibiotic; %S: Percentage of susceptible isolates;

N: total number of isolates; *: less than 40% susceptibility; boldened :69% – 40% susceptibility; italicized : 70% and above susceptibility

Table S3: Resistance profiles of epidemiologically significant pathogens

| Organism | n | Antimicrobial agent | Number of isolates tested | %R | %I | %S |
| --- | --- | --- | --- | --- | --- | --- |
| *E. coli* | 246 | Piperacillin | 61 | 96.7 | 1.6 | 1.6 |
|  |  | Ampicillin | 26 | 84.6 | 3.8 | 11.5 |
|  |  | Ceftazidime | 67 | 77.6 | 3.0 | 19.4 |
|  |  | Ceftizoxime | 25 | 68.0 | 0.0 | 32.0 |
|  |  | Nalidixic acid | 102 | 67.6 | 3.9 | 28.4 |
|  |  | Ceftriaxone | 85 | 67.1 | 7.1 | 25.9 |
|  |  | Tetracycline | 99 | 64.6 | 2.0 | 33.3 |
|  |  | Moxifloxacin | 32 | 62.5 | 0.0 | 37.5 |
|  |  | Ciprofloxacin | 172 | 59.9 | 2.9 | 37.2 |
|  |  | Norfloxacin | 44 | 54.5 | 0.0 | 45.5 |
|  |  | Cefuroxime | 50 | 52.0 | 4.0 | 44.0 |
|  |  | Cefuroxime | 50 | 52.0 | 4.0 | 44.0 |
|  |  | Gentamicin | 184 | 41.8 | 2.2 | 56.0 |
|  |  | Nitrofurantoin | 118 | 34.7 | 8.5 | 56.8 |
|  |  | Levofloxacin | 149 | 14.8 | 6.7 | 78.5 |
|  |  | Netilmicin | 34 | 14.7 | 8.8 | 76.5 |
|  |  | Amikacin | 176 | 3.4 | 0.0 | 96.6 |
|  |  |  |  |  |  |  |
| *Pseudomonas aeruginosa* | 114 | Ampicillin | 23 | 87 | 8.7 | 4.3 |
|  |  | Colistin | 24 | 70.8 | 16.7 | 12.5 |
|  |  | Gentamicin | 26 | 65.4 | 7.7 | 26.9 |
|  |  | Ciprofloxacin | 24 | 58.3 | 0 | 41.7 |
|  |  | Levofloxacin | 29 | 44.8 | 6.9 | 48.3 |
|  |  | Ceftizoxime | 23 | 34.8 | 4.3 | 60.9 |
|  |  | Amikacin | 28 | 14.3 | 7.1 | 78.6 |
|  |  | Moxifloxacin | 36 | 11.1 | 2.8 | 86.1 |
|  |  | Netilmicin | 62 | 6.5 | 9.7 | 83.9 |
|  |  | Lomefloxacin | 28 | 3.6 | 0 | 96.4 |
|  |  |  |  |  |  |  |
| *Klebsiella oxytoca* | 54 | Ciprofloxacin | 37 | 73 | 5.4 | 21.6 |
|  |  | Gentamicin | 40 | 57.5 | 0 | 42.5 |
|  |  | Nitrofurantoin | 24 | 41.7 | 12.5 | 45.8 |
|  |  | Levofloxacin | 27 | 14.8 | 14.8 | 70.4 |
|  |  | Amikacin | 33 | 9.1 | 0 | 90.9 |
|  |  |  |  |  |  |  |
| *Citrobacter koseri* | 34 | Tetracycline | 13 | 69.2 | 0 | 30.8 |
|  |  | Ciprofloxacin | 20 | 65 | 5 | 30 |
|  |  | Moxifloxacin | 12 | 58.3 | 16.7 | 25 |
|  |  | Gentamicin-High | 19 | 47.4 | 5.3 | 47.4 |
|  |  | Netilmicin | 11 | 36.4 | 0 | 63.6 |
|  |  | Nitrofurantoin | 14 | 21.4 | 14.3 | 64.3 |
|  |  | Levofloxacin | 17 | 11.8 | 17.6 | 70.6 |
|  |  |  |  |  |  |  |
| *Morganella morganii* | 5 | Ceftizoxime | 4 | 50.0 | 0.0 | 50.0 |
|  |  | Chloramphenicol | 2 | 50.0 | 0.0 | 50.0 |
|  |  | Colistin | 2 | 0.0 | 50.0 | 50.0 |
|  |  | Gemifloxacin | 3 | 66.7 | 0.0 | 33.3 |
|  |  | Moxifloxacin | 4 | 25.0 | 0.0 | 75.0 |
|  |  | Nalidixic acid | 3 | 33.3 | 33.3 | 33.3 |
|  |  | Netilmicin | 5 | 40.0 | 0.0 | 60.0 |
|  |  |  |  |  |  |  |
| *Moraxella catarrhalis* | 5 | Aztreonam | 3 | 0.0 | 0.0 | 100.0 |
|  |  | Cefoxitin | 3 | 33.3 | 0.0 | 66.7 |
|  |  | Ceftizoxime | 3 | 0.0 | 0.0 | 100.0 |
|  |  | Moxifloxacin | 4 | 75.0 | 0.0 | 25.0 |
|  |  | Netilmicin | 2 | 50.0 | 0.0 | 50.0 |
|  |  | Penicillin G | 3 | 100.0 | 0.0 | 0.0 |
|  |  |  |  |  |  |  |
| *Proteus mirabilis* | 12 | Amikacin | 3 | 0.0 | 0.0 | 100.0 |
|  |  | Ampicillin | 3 | 66.7 | 0.0 | 33.3 |
|  |  | Aztreonam | 2 | 50.0 | 0.0 | 50.0 |
|  |  | Cefixime | 2 | 0.0 | 0.0 | 100.0 |
|  |  | Ceftazidime | 2 | 50.0 | 0.0 | 50.0 |
|  |  | Ceftizoxime | 3 | 33.3 | 0.0 | 66.7 |
|  |  | Cefuroxime | 2 | 0.0 | 0.0 | 100.0 |
|  |  |  |  |  |  |  |
| *Proteus vulgaris* | 13 | Ciprofloxacin | 3 | 100.0 | 0.0 | 0.0 |
|  |  | Colistin | 4 | 25.0 | 0.0 | 75.0 |
|  |  | Gemifloxacin | 2 | 0.0 | 0.0 | 100.0 |
|  |  | Gentamicin-High | 4 | 25.0 | 0.0 | 75.0 |
|  |  | Levofloxacin | 2 | 0.0 | 0.0 | 100.0 |
|  |  | Moxifloxacin | 8 | 25.0 | 0.0 | 75.0 |
|  |  | Netilmicin | 8 | 25.0 | 12.5 | 62.5 |
|  |  | Tetracycline | 2 | 0.0 | 0.0 | 100.0 |
|  |  | Cefuroxime | 2 | 0.0 | 0.0 | 100.0 |
|  |  |  |  |  |  |  |
| *Proteus mirabilis* | 12 | Ampicillin | 4 | 50.0 | 25.0 | 25.0 |
|  |  | Azithromycin | 2 | 50.0 | 0.0 | 50.0 |
|  |  | Aztreonam | 5 | 40.0 | 0.0 | 60.0 |
|  |  | Cefixime | 2 | 50.0 | 0.0 | 50.0 |
|  |  | Cefoxitin | 2 | 50.0 | 50.0 | 0.0 |
|  |  | Ceftizoxime | 2 | 50.0 | 0.0 | 50.0 |
|  |  | Chloramphenicol | 2 | 0.0 | 0.0 | 100.0 |
|  |  | Ciprofloxacin | 2 | 50.0 | 0.0 | 50.0 |
|  |  | Colistin | 5 | 40.0 | 20.0 | 40.0 |
|  |  |  |  |  |  |  |
| *Klebsiella pneumoniae* | 12 | Amikacin | 2 | 0.0 | 0.0 | 100.0 |
|  |  | Amoxicillin/Clavulanic acid | 2 | 50.0 | 0.0 | 50.0 |
|  |  | Ampicillin | 4 | 75.0 | 0.0 | 25.0 |
|  |  | Azithromycin | 2 | 0.0 | 0.0 | 100.0 |
|  |  | Cefixime | 3 | 33.3 | 0.0 | 66.7 |
|  |  | Ceftizoxime | 2 | 50.0 | 50.0 | 0.0 |
|  |  | Ceftriaxone | 2 | 100.0 | 0.0 | 0.0 |
|  |  | Cefuroxime | 2 | 100.0 | 0.0 | 0.0 |
|  |  | Chloramphenicol | 3 | 66.7 | 0.0 | 33.3 |
|  |  | Ciprofloxacin | 2 | 0.0 | 0.0 | 100.0 |
|  |  | Colistin | 4 | 25.0 | 0.0 | 75.0 |
|  |  | Gemifloxacin | 3 | 0.0 | 0.0 | 100.0 |
|  |  | Gentamicin-High | 2 | 50.0 | 0.0 | 50.0 |
|  |  | Levofloxacin | 2 | 0.0 | 0.0 | 100.0 |
|  |  | Moxifloxacin | 7 | 0.0 | 0.0 | 100.0 |
|  |  | Nalidixic acid | 3 | 33.3 | 0.0 | 66.7 |
|  |  | Netilmicin | 6 | 33.3 | 0.0 | 66.7 |
|  |  | Tetracycline | 2 | 50.0 | 0.0 | 50.0 |
|  |  | Cefuroxime | 2 | 100.0 | 0.0 | 0.0 |
|  |  |  |  |  |  |  |
| *Staphylococcus aureus* | 49 | Amoxicillin | 3 | 100.0 | 0.0 | 0.0 |
|  |  | Ampicillin | 39 | 66.7 | 0.0 | 33.3 |
|  |  | Cefixime | 23 | 87.0 | 4.3 | 8.7 |
|  |  | Chloramphenicol | 22 | 27.3 | 0.0 | 72.7 |
|  |  | Ciprofloxacin | 4 | 25.0 | 0.0 | 75.0 |
|  |  | Colistin | 22 | 59.1 | 13.6 | 27.3 |
|  |  | Doxycycline | 37 | 24.3 | 5.4 | 70.3 |
|  |  | Erythromycin | 3 | 66.7 | 33.3 | 0.0 |
|  |  | Gentamicin-High | 4 | 25.0 | 25.0 | 50.0 |
|  |  | Moxifloxacin | 39 | 7.7 | 10.3 | 82.1 |
|  |  | Netilmicin | 36 | 13.9 | 11.1 | 75.0 |
|  |  | Penicillin G | 28 | 85.7 | 7.1 | 7.1 |
|  |  | Tetracycline | 4 | 75.0 | 25.0 | 0.0 |
|  |  | Trimethoprim/Sulfamethoxazole | 2 | 100.0 | 0.0 | 0.0 |
